# Role of Naltrexone in Improving Compulsive Drinking in Psychogenic Polydipsia

**DOI:** 10.7759/cureus.5320

**Published:** 2019-08-05

**Authors:** Sukaina Rizvi, Jeffrey Gold, Ali M Khan

**Affiliations:** 1 Psychiatry, Manhattan Psychiatric Center, Manhattan, USA; 2 Psychiatric, Manhattan Psychiatric Center, Manhattan, USA; 3 Psychiatry, University of Texas Rio Grande Valley, Harlingen, USA

**Keywords:** naltrexone, psychogenic polydipsia, schizophrenia, compulsive drinking, opioid receptor

## Abstract

Psychogenic polydipsia or self-induced water intoxication is a potentially lethal condition seen in many chronic psychiatric patients. This is a literature review based on therapeutic significance of Naltrexone in improving compulsive water drinking behavior in chronic psychiatrically ill patients with psychogenic polydipsia. Naltrexone is an opioid antagonist approved by FDA for alcohol dependence. Extensive literature search provides a line of evidence that suggests correlation of opioid receptor with compulsive water ingestion in animals. However, there is limited data regarding clinical utility of naltrexone in improving psychogenic polydipsia in human species. This review highlights the necessity for further research and trials to elucidate the role of naltrexone in human psychogenic drinking behavior.

## Introduction and background

Psychogenic polydipsia (PP) is a chronic episodic condition in psychiatric patients characterized by compulsive drinking of large amounts of water resulting in dilutional hyponatremia and various neurological signs. It is estimated that 15% to 25% of patients with chronic mental disease have psychogenic polydipsia [1,2,3]. Schizophrenia is the most commonly associated psychiatric disorder with 80% of cases reported with PP [1,2]. The other psychiatric disorders linked to PP are anorexia nervosa, psychotic depression, and bipolar depression [4,5,6].

Psychogenic polydipsia is a three-phase process. First, polydipsia is accompanied by polyuria, followed by hyponatremia; finally there are occurrences of neurological signs such as delirium, ataxia, seizures and coma secondary to ingestion of excessive quantity of water [3].

## Review

Psychogenic polydipsia or self-induced water intoxication is a relatively common condition in chronic psychiatric patients, occurring from 5 to 10 years after the onset of psychosis. It presents as a chronic condition in 67% of cases, and episodic in 33% of cases [1]. It is defined by the consumption of more than 3 liters of fluids per day with excretion of profound amount of dilute urine. Hyponatremia is observed to co-occur in 30% cases of PP [1,7]. However, only one-third to one-fifth of the patients with PP present with symptomatic hyponatremia. PP in schizophrenics drastically affects the morbidity, mortality and disease prognosis leading to reduction in life expectancy by 13% [1,8]. The exact etiology of PP is idiopathic as multiple factors may play a role in its pathogenesis.

Multiple analyses have suggested race (male Caucasians), smoking, and chronic schizophrenia to be the strongest risk factors associated with PP [1,3,9]. Moreover, research studies have speculated that PP with water intoxication occurs more commonly in patients with negative symptoms of schizophrenia [1,10]. Similarly, hyponatremia in PP was more often observed in institutionalized psychiatric patients with greater treatment resistance which can contribute to cognitive decline and delay in processing information [11,12]. Poirier et al attributed alcohol addiction as a risk factor for development of water intoxication in PP patients [4,13].

The management of PP has always been a challenge for psychiatrists, as there is no definitive treatment despite its universal existence in many long-term psychiatric facilities. Over the years, psychiatrists have implemented techniques such as behavior modification and psychosocial rehabilitation for effective management. Studies have also hypothesized the possible role of atypical anti-psychotics such as clozapine, olanzapine, and risperidone in improving the anxiety related to self-drinking behavior [14,15,16]. However, there are varied theories regarding their efficacy in improving compulsive drinking, as hyponatremia secondary to olanzapine can further exacerbate the symptoms of psychosis. Animal studies have also suggested the involvement of the alpha2- adrenergic system in mediating the excessive drinking behavior in chronic schizophrenics [17].

Naltrexone, an opiate antagonist, is approved by Food and Drug Administration to treat opioid use disorder and alcohol use disorder. However, over the years, research and molecular studies have diversified our knowledge regarding implication of opiate receptors. Based on opiate receptor and its complex interactions, there could be a possibility where naltrexone can play a significant role in improving self-drinking behaviors. Our understanding regarding naltrexone’s clinical significance supports the idea by Snyder in the year 1984, where they correlated the consequences of polydipsia on opiate receptors like addictive behaviors [18,19]. The stereotypic behaviors in PP patients shared some similarity to behaviors observed in Lesch-Nyan syndrome and mentally challenged patients where they showed diminished symptoms after treatment with an opiate antagonist. This led Nishikawa et al to speculate the role of naloxone improving PP in some schizophrenics [19,20].

Multiple animal studies have postulated that blockade of opioid neurotransmission leads to reduction in body weight and thirst mechanism. Opioid peptides and receptors are highly condensed in paraventricular and supraoptic hypothalamic nuclei which are believed to be the site of opioid antagonism in animals [21]. Kurbanovb et al studied the effects of naltrexone on food intake and body weight in olanzapine treated rats. They observed significant reduction in food intake and body weight in the combined olanzapine and naltrexone treated group in contrast to the group that only received naltrexone [22].

In an open design study, naltrexone 50 mg PO/day was added for a period of 6-weeks to the anti-psychotic regimen of seven inpatient PP hyponatremic patients where they demonstrated significant reduction in mannerisms and diurnal weight change during their last treatment period. However, the results of the study were limited due to small sample size and warranted larger studies to support the hypothesis that behaviors such as compulsive drinking were mediated by endogenous opiates [19,23]. More recently, a successfully treated case of PP has been reported where a 47-year old male patient with PP responded well to a combination therapy of irbesartan 300 mg and naltrexone 50 mg [24].

## Conclusions

Psychogenic polydipsia is a common condition observed in various psychiatric facilities. Fluid restriction is a successful measure to treat the acute symptoms. However, there is a need for pharmacological intervention to treat the polydipsic symptoms in the long term. Given the above-mentioned facts, it is implied that naltrexone can play an pivotal role in treating psychogenic polydipsia in chronic psychiatric patients. However, more cases should be reported to elaborate the efficacy of naltrexone as an adjuvant in treating self-drinking behaviors.
